# The Fate of Epidermal Tight Junctions in the *stratum corneum*: Their Involvement in the Regulation of Desquamation and Phenotypic Expression of Certain Skin Conditions

**DOI:** 10.3390/ijms23137486

**Published:** 2022-07-05

**Authors:** Marek Haftek, Vinzenz Oji, Laurence Feldmeyer, Daniel Hohl, Smaïl Hadj-Rabia, Rawad Abdayem

**Affiliations:** 1 UMR5305, Laboratory of Tissue Biology and Therapeutic Engineering, CNRS and University of Lyon, 8 Avenue Rockefeller, F-69373 Lyon, France; 2 Department of Dermatology, University Hospital of Muenster, D-48149 Muenster, Germany; vinzenz.oji@ukmuenster.de; 3 Department of Dermatology, University Hospital Lausanne, CH-1011 Lausanne, Switzerland; daniel.hohl@chuv.ch; 4 Department of Dermatology, Reference Center for Genodermatoses and Rare Skin Diseases (MAGEC), Institut Imagine, Necker-Enfants Malades Hospital, AP-HP5, University Paris-Centre, F-75015 Paris, France; smail.hadj@inserm.fr

**Keywords:** tight junction, *stratum corneum*, desquamation, peeling skin disease (PSD), ichthyosis hypotrichosis sclerosing cholangitis (IHSC/NISCH) syndrome, ultrastructure

## Abstract

We evaluated the presence of tight junction (TJ) remnants in the *stratum corneum* (SC) of in vitro reconstructed human epidermis and human skin explants subjected or not to an aggressive topical treatment with beta-lipohydroxy salicylic acid (LSA) for 24 h. LSA-treated samples showed an increased presence of TJ remnants in the two lowermost layers of the SC, as quantified with standard electron microscopy. The topical aggression-induced overexpression of TJ-like cell–cell envelope fusions may influence SC functions: (1) directly, through an enhanced cohesion, and (2) indirectly, by impeding accessibility of peripheral corneodesmosomes to extracellular hydrolytic enzymes and, thus, slowing down desquamation. Observations of ichthyotic epidermis in peeling skin disease (PSD; corneodesmosin deficiency; two cases) and ichthyosis hypotrichosis sclerosing cholangitis syndrome (IHSC/NISCH; absence of claudin-1; two cases) also demonstrated increased persistence of TJ-like intercellular fusions in pathological SC and contributed to the interpretation of the diseases’ pathological mechanisms.

## 1. Introduction

The *stratum corneum* (SC) is the final product of the process of epidermal differentiation. Besides its crucial protective role as a physical permeability barrier, this composite structure made of cornified keratinocytes embedded in the layered lipid matrix is also, by nature, a tissue that holds “memory” of the past events occurring in the subjacent living layers. In normal human epidermis, the cornification process is very rapid. It takes approximately 14 h for the uppermost granular layer of keratinocytes to degrade their nuclei and cytoplasmic organelle, massively extrude the contents of lamellar granules, and flatten out and replace the plasma membranes with cornified lipid envelopes. In the process, several structures expressed by the living cells become entrapped and immobilized at the cells’ periphery. Cell–cell junctions are obvious targets of transglutaminases that cross-link junctions’ components within the corneocyte envelopes. Thus, desmosomes and tight junctions (TJs) of the living cells become fixed on the cornified cells and cannot be recycled anymore. It is possible to quantify the structures observed ultrastructurally in the lower SC under experimental conditions or in pathological situations and to draw conclusions on the living tissue dynamics over several hours [1]. A schematic illustration of the process of terminal differentiation of epidermal keratinocytes and involvement of cell–cell junctions therein is presented in the Supplementary Figure S1.

Tight junctions belong to the family of intercellular junctions that provide very close contact between cell membranes of interacting cells [2,3]. In simple epithelia, where TJs form continuous bands around the apical portions of the cells, the lumen of secretory ducts and cavities becomes efficiently isolated from the body’s physiological internal milieu. As in most of the cell–cell junctions, the TJ structure is based on transmembrane proteins (occludin, several claudins, JAM-A, MUPP-1, etc.) that interact in the extracellular space, are stabilized by the intracellular subplasmalemmal junction proteins (ZO-1, cingulin), and become attached through the latter to the contractile actin cytoskeleton [4,5]. TJ strands encircling cell apices subdivide the cell surface into two domains, the apical and basolateral, resulting in cell polarization and allowing for pole-specific activities. In fully functional TJs, the extracellular leaflets of interacting plasma membranes become so closely apposed that there is no more extracellular space observable ultrastructurally. Perfusion of studied tissues with tracers of various molecular sizes helps to reveal the sites where paracellular diffusion is no longer possible, thus defining the presence of the TJ barrier [6]. However, “tightness” of the junctions depends largely on their protein composition and, in some instances, TJs may allow paracellular passage of water and ions. 

In human epidermis, the presence of TJs was first described by Ken Hashimoto, who observed focal membrane fusions between the apical parts of adjacent keratinocytes in the uppermost granular layer just before cell cornification [7]. The structures were ephemeral but corresponded to the sites of the “tracer stop” in that ultrastructural study, which was a solution of lanthanum salts. Unfortunately for the further understanding of the role that TJs could play in the epidermis, the discovery of structured lamellar lipids secreted into the extracellular space by granular layer keratinocytes upon formation of the SC almost completely eclipsed Hashimoto’s findings. It became the dogma, still currently held, that hydrophobicity of the SC is the principal factor providing the epidermal permeability barrier [8,9,10,11]. Thirty years after Hashimoto’s first description, progress in genetic, biochemical, and visualization techniques has sparked renewed interest in TJ research. In their seminal study in mice, Furuse et al. demonstrated that genetically invalidated expression of claudin-1 (Cldn1) deprived new-born animals of an efficient skin barrier function, provoking rapid death from dehydration [12]. Since then, several studies have been undertaken to better understand the way TJs may influence the constitution and function of the epidermal permeability barrier [13]. In human epidermis and hair follicles, the presence of TJs in the granular layer (*stratum granulosum*, SG) could be confirmed by immuno-histochemical and ultrastructural studies using antibodies against proteins that are specific to the functional junctions and by “tracer stop” assays, respectively [6,14,15,16,17]. It has been proposed that continuous belts of functional TJs are formed between the SG keratinocytes, at the level of two cell layers before cornification occurs; the SG layers situated below and above this level present only fragmentary stretches of TJs [16]. Based on the functional evidence of existence of the TJ barrier in the living part of human epidermis, additional questions need to be addressed. These focus principally on the plausibility of an interaction between TJs and SC in the formation and maintenance of the functional permeability barrier in normal and diseased human skin [16,18,19,20,21].

We and others have previously confirmed the presence of TJs, visualized ultrastructurally and reactive with antibodies to TJ proteins, in the SG of human skin [22,23,24]. Remarkably, TJs visualized ultrastructurally in human epidermis were localized predominantly at the apical-lateral walls of SG keratinocytes, as previously reported by Hashimoto [7]. Furthermore, we demonstrated the persistence of TJ-like fusions between the corneocyte envelopes at a similar localization [1,22,23], suggesting entrapment of the pre-existing TJ structures by cross-linking in the course of SC formation [25]. Corneocyte envelopes, purified using extensive physicochemical protein extraction, retained such points of fusion reactive with anti-Cldn1 antibodies [23]. Additionally, TJ remnants in the SC could be immunolabeled with antibodies to occludin and Cldn1 on ultracryo sections of normal human epidermis, whereas proteolytic kallikrein 7 was localized to the inter-corneocyte spaces between the consecutive layers of the SC [24].

In the present study we searched for clues indicating an interrelationship between the TJ and SC barriers in human epidermis using a quantitative ultrastructural approach [1].

## 2. Results

### 2.1. Visualization and Quantitation of TJ-Derived Points of Fusion between Corneocytes in the SC Compactum of Normal Human Skin (NHS)

**Figure 1:**
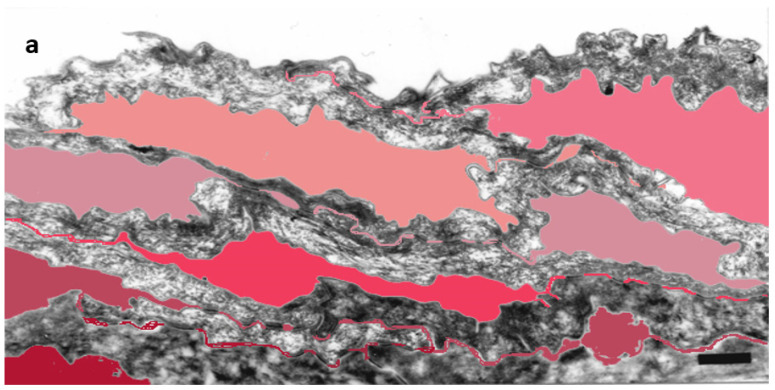

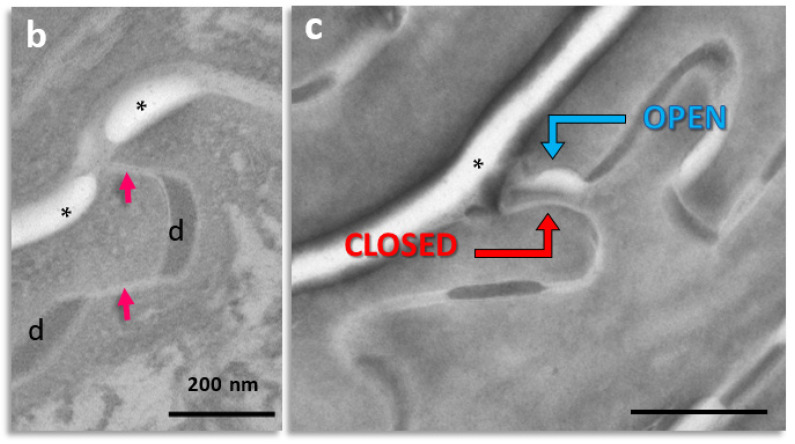
Subdivision of the extracellular space of SC into separate compartments, (**a**) indicated in different colors; (**b**) apical-lateral TJ remnants (arrows) intercalating corneodesmosomes (d) may be quantified; (**c**) an example of “closed” and “open” apical-lateral contacts between the three SC 2 corneocytes. TJ-derived fusions of corneocyte envelopes seal the extracellular space (*). Bar in (**a**) = 1 µm; in (**b**,**c**) = 200 nm.

Detailed ultrastructural observations confirmed that TJ-derived points of contact persist in the SC (Figure 1) and can be quantified (Table 1). 

Significant upregulation of the apical-lateral TJ-derived fusion of corneocyte envelopes was observed in the skin explants maintained 24h in survival conditions (open or closed chambers at 37 °C) when compared to fresh skin biopsies. The major difference between these two situations is that the fresh skin biopsies were chemically fixed immediately after excision, whereas skin explants underwent the fixation procedure only after being “cultured”, which allowed for additional time of SC formation under experimental conditions.

To eliminate the possibility that the observed differences were dependent on the anatomical region of skin origin, i.e., extensor surface of the arm vs. lower abdomen in middle-aged women, an additional study was conducted. Comparison of the TJ-derived structure frequency in the SC compactum between biopsies taken from the two anatomical sites revealed no body region-dependent differences (51.4 ± 10.1% for arm vs. 51.5 ± 4.7% for abdomen; *n* = 7). Subsequently, no attention was paid to biopsy site in further studies.

The recorded fluctuations in the number of TJ-derived fusions could correspond to dynamic changes in TJ expression occurring in the underlying living epidermal layers (Table 1). 

### 2.2. Quantitation of Tight Junction Remnants in SC Reveals a Highly Interactive Line of Protection against Environmental Aggression

Salicylic acid is a beta hydroxy acid, also classified as a phenolic aromatic acid, that has multifunctional uses in the treatment of various skin conditions such as ichthyosis, acne, psoriasis, and photoaging [26]. It presents colliquation properties and mainly interferes with molecular interactions in the intercorneocyte space, dissolving corneodesmosomal junctions and destabilizing the intercellular lipid matrix [27]. A lipophilic derivative of salicylic acid (LSA), also known in the literature as 2-hydroxy-5-octanoyl benzoic acid, beta-lipohydroxy acid, or capryloyl salicylic acid is used as an exfoliant and as a treatment for photoaged skin and acne [28]. Its C8 polycarbon tail facilitates integration in the intercellular lipid matrix of the SC and promotes retention of the compound in the targeted horny layer and, therefore, keratolysis. In a freeze-fracture and transmission electron microscopy study of NHS treated ex vivo with topical LSA, cleavage of corneodesmosome cores followed by dissociation of the SC have been observed [28]. The treatment also provoked an occurrence of typical linear strands of TJs in the SC compactum of reconstructed human epidermis (RHE) exposed to LSA (unpublished observations of F. Fiat and A-M. Minondo). This fact suggests the possibility of reactive upregulation of epidermal TJs in response to the disruption of the SC barrier.

To test this hypothesis, excised human skin (abdomen) maintained ex vivo in survival conditions and in vitro cultured epidermis reconstructed out of normal human keratinocytes, RHE (EpiSkin^®^, Episkin, Lyon, France), were exposed to topically applied 1% LSA (L’Oréal, Aulnay sous Bois, Paris, France) vs. a vehicle (propylene glycol) for 24 h at 37 °C. The samples were processed through standard electron microscopy and TJ-like fusions situated at the apical-lateral portions of corneocytes were quantified in the SC compactum (Table 2).

In both in vitro (RHE) and ex vivo (explants) settings, only the two first cornified layers above the SG were evaluated. Because the cornification rate in normal human epidermis may be estimated at 1.7 freshly cornified cells per 24 h, quantitation of TJ remnants in the lowermost SC (SC compactum) should correspond to the newly cross-linked junctions.

A significant increase in TJ-derived fusions within the apical-lateral contacts of corneocytes was observed in the first two newly cornified layers of the SC in the in vitro cultured RHE, but not in the native skin maintained in survival conditions, suggesting a better resistance of the latter to a topical chemical challenge. 

### 2.3. Regulation of SC Cohesion/Desquamation by Persistent TJ Remnants in Skin Pathology

TJs show aberrant expression and function in numerous skin diseases, e.g., psoriasis, atopic dermatitis, and rosacea [5,21,29,30,31,32,33,34,35,36]. Here, we have focused on the potential role of the physical rivets provided by TJ-like fusions in pathological SC of two ichthyotic skin conditions.

#### 2.3.1. Tight Junction Remnants Contribute to the Compensatory Hyperkeratosis in Cldn1-Deficient Patients with Ichthyosis Hypotrichosis Sclerosing Cholangitis (IHSC) Syndrome

A transmembrane TJ component, Cldn1, has an important impact on the junction’s function. As demonstrated in Cldn1 deficient mice, this protein is essential for TJ water barrier function, as KO animals die rapidly after birth from dehydration [12]. However, human natural Cldn-1^−/−^ mutants survive the neonatal period and develop a syndromic ichthyosis (NISCH/IHSC syndrome) [37,38] (Supplementary Figure S2).

**Figure 2:**
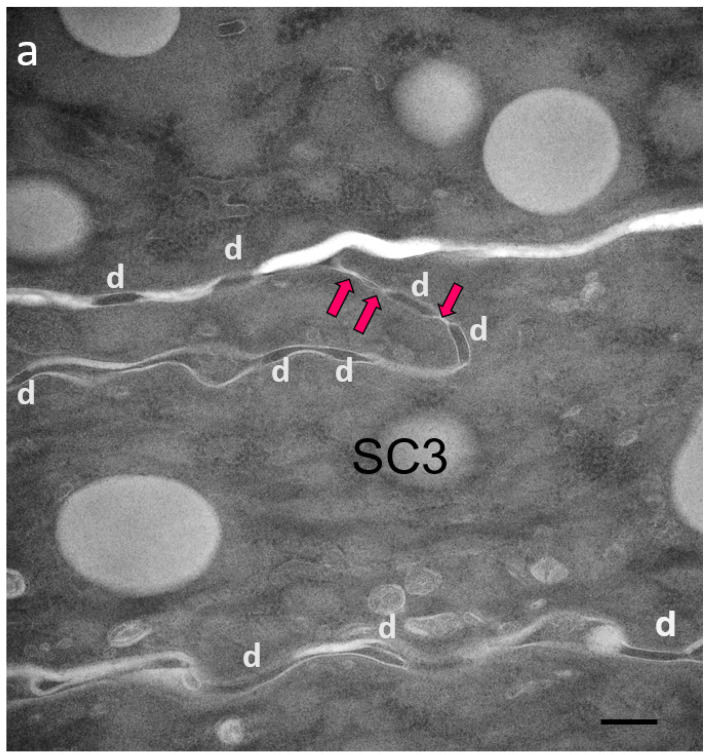

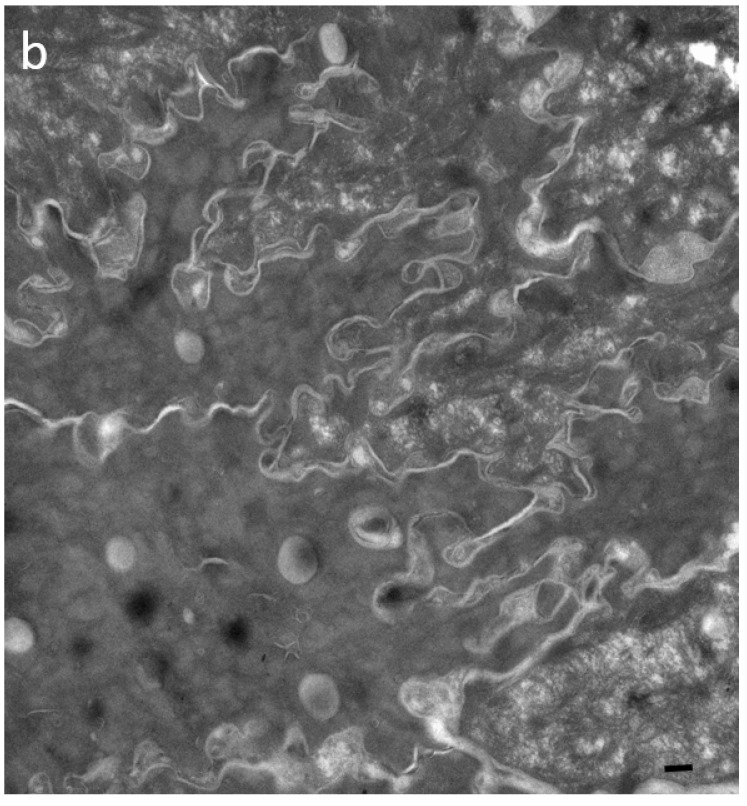

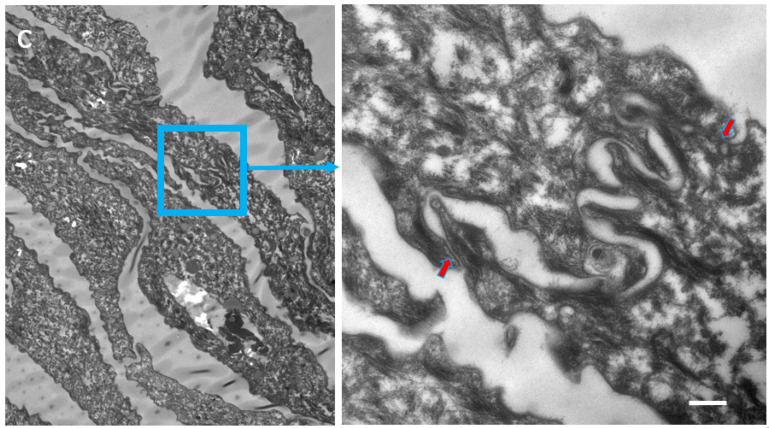
Ultrastructural features of SC in patients with IHSC include problems related to lipid extrusion (**a**), abnormal convolution of cell walls, and increased retention of TJ-derived intercorneocyte rivets (**b**,**c**). TJ-derived fusion points between the lateral walls of cornified cells persist up to the surface of hyperkeratotic SC, shown in (**c**), where corneodesmosomes are no longer present. Arrows point to TJ remnants isolating the extracellular space between adjacent uppermost corneocytes. In (**c**), the right panel shows a high power view of the SC disjunctum area delimited by the rectangle to the left. d = (corneo)desmosome; red arrows = TJ-derived structure. Bars = 200 nm.

We proposed the hypothesis that in Cldn1 deficient human skin, reactive hyperkeratosis may compensate for defective TJ function. Contrary to the mouse SC which also shows hyperkeratosis in the absence of Cldn1, the horny layer in humans appears to be better fit for providing the barrier function (Figure 2a–c; Table 3). 

Two patients with IHSC syndrome were examined: one bearing a homozygous deletion (200delTT) in the first exon of the *CLDN1* gene, resulting in a premature stop codon [37], the other a *CLDN1* frameshift mutation in exon 2 [38]. Both showed a total absence of the protein. Morphological examination of IHSC biopsies revealed acanthosis, focal parakeratosis, and papillomatosis characteristic of an epidermal proliferative response. Partial retention of lamellar granules and numerous lipid vacuoles in the SC could be seen ultrastructurally. Highly convoluted lateral walls of corneocytes with narrow intercellular spaces and numerous TJ-derived contacts persisted throughout the thickened SC. 

**Figure 3:**
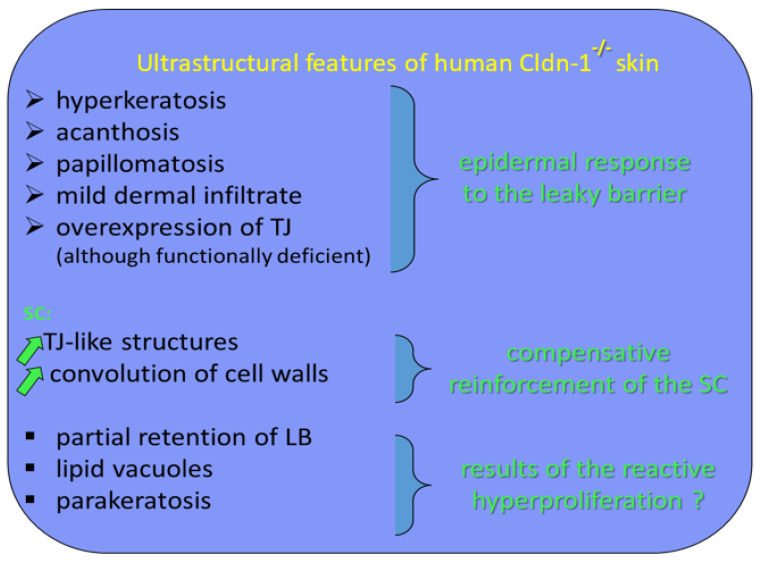
Ultrastructural features of human Cldn-1^−/−^ skin and their functional translation.

We suggest that the TJ remnants persisting between SC corneocytes contribute to cohesion of the horny layer. As a consequence of this thesis, in human epidermis, a leaky TJ barrier could be compensated by hyperkeratosis through the mechanism of increased expression of TJs, which would be functionally inefficient in terms of barrier function but would be still cross-linked during cornification. It appears that in our patients with IHSC syndrome, the SC retention and, thus, hyperkeratosis are linked to an overexpression of TJ-like structures. Therefore, TJs may also influence epidermal barrier function in a manner independent of their molecular composition. Although the SC is the primary permeability barrier of human epidermis, its formation may be regulated by TJs (Figure 3).

Acanthosis is a benign, abnormal thickening of the SS; parakeratosis is a retention of nuclei in the cells of the SC; and papillomatosis consists of papillary projections of the epidermis into the dermis forming a microscopically undulating surface.

#### 2.3.2. Ultrastructural Evaluation of the *stratum corneum* in Peeling Skin Disease (PSD) Suggests a Compensatory TJ Upregulation

Peeling skin disease (PSD) was the first genodermatosis described to be caused by mutations in the corneodesmosin (*CDSN*) gene [39]. PSD is characterized by premature detachment of the SC due to corneodesmosome fragility. Interestingly, the mechanically induced split primarily localizes to the interface between the SG and SC but, curiously, cohesion of the upper SC seems to be stabilized. This results in a clinical picture of flaky detachment of SC sheets (Supplementary Figure S3). Both PSD patients included in the present study showed the same homozygous nonsense mutation in *CDSN*, c.175A>T, resulting in a premature termination codon, p.Lys59X.

We hypothesized that diminished corneodesmosome-mediated cohesion might be partially compensated for by overexpression of another type of intercellular junction persisting in the SC, the modified TJ. 

**Figure 4:**
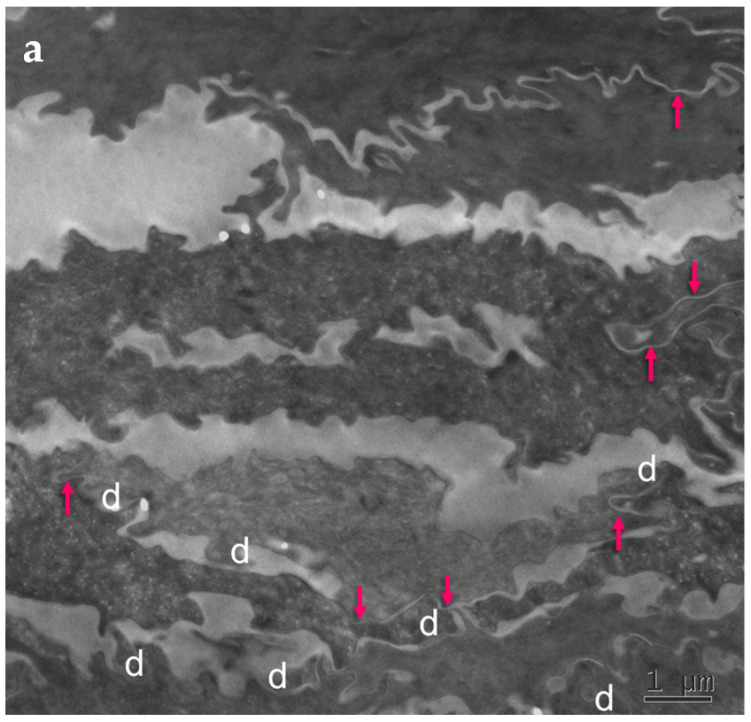

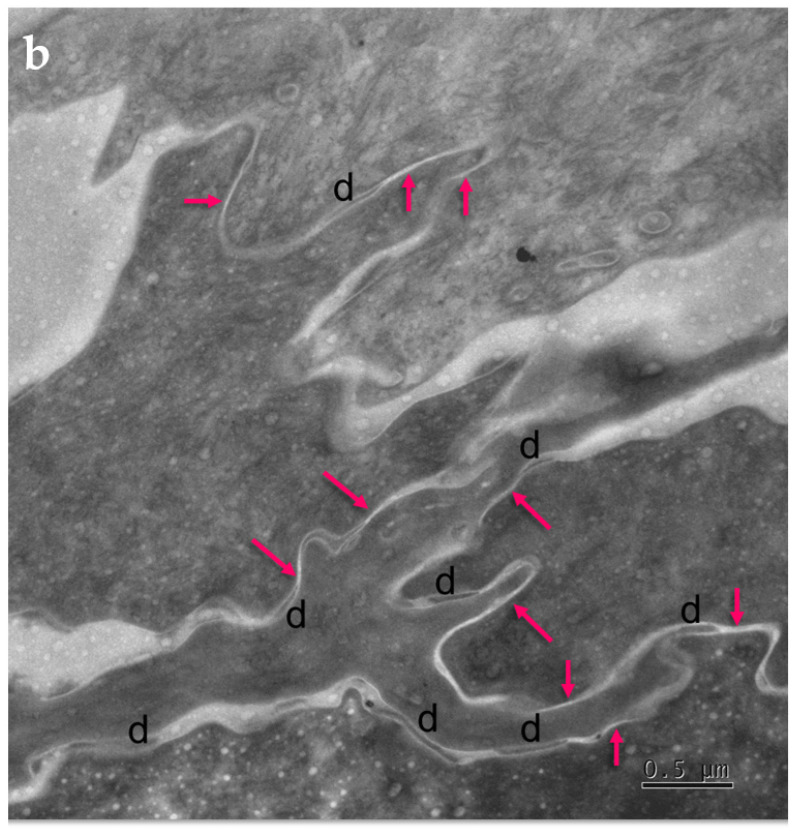
Ultrastructural features observed in the SC of PSD include paucity of corneodesmosomes (d) that rapidly disappear from the lower parts of the horny layer, as seen in (**a**), and upregulation of expression of TJ-derived intercorneocyte “fusions” (arrows). (**b**) In the upper SC, the remaining corneodesmosomes (d) are often found in the proximity of the TJ-derived riveting structures (arrows). Bars = 1 µm in (**a**); 0.5 µm in (**b**).

Skin biopsies and specimens of peeled-off SC were obtained from two PSD patients (Supplementary Figure S3) and examined with standard electron microscopy (Figure 4). Numerous, long TJ remnants showing “fused” lipid envelope morphology were present in PSD and contributed to the lateral cohesion within the SC. They also seemed to promote formation of highly developed interdigitations, which constituted yet another factor rescuing SC cohesiveness despite degradation of “central” corneodesmosomes situated between consecutive layers of corneocytes.

The cell–cell fusions of lipid envelopes in the upper part of the lateral intercorneocyte contacts, which have been shown to result from the persistence of TJ structures, were quantified and the results compared with the situation in 35 normal controls (Table 4).

Upregulation of the TJ-derived corneocyte envelope fusions in PSD may represent a compensatory phenomenon in face of corneodesmosome fragility. It may contribute to cohesion in the upper SC directly, through enhanced cell–cell adhesion, and indirectly, by hindering access of extracellular hydrolytic enzymes to corneodesmosomes.

Mature upper SC is more rigid than living epidermis and lower, freshly cornified keratinocytes [40]. In this situation, minor mechanical insults to tissue lacking reinforced corneodesmosomes may provoke a preferential split at the interface between the two parts. The upper part of the horny layer of PSD appears to be reinforced by the increased presence of cross-linked TJ remnants.

## 3. Discussion

TJ-derived points of contact persist in the SC, and the TJ protein elements (e.g., Cldn1 and occludin) become trapped in cornified cell envelopes [22,23,24]. Observation and quantitation of these structures in various conditions of epidermal development indicate significant variation, testifying to the dynamic TJ changes occurring during SC formation in vivo and in vitro.

Indeed, total or partial inhibition of expression of key TJ proteins has been shown to clearly impact epidermal permeability by influencing filaggrin and lipid processing and by leading to abnormal formation of SC [41]. Additionally, SC barrier formation is impaired in atopic dermatitis (AD) skin—a fact that coincides with the reported alteration of Cldn-1 expression and TJ defects in this disease [30,32,42]. Because a substantial number of AD cases are due to loss-of-function mutations in the filaggrin gene, it is likely that the observed inter-relation between the two barriers is reciprocal. The observation that the formation of the SC barrier may be modulated experimentally by changes in the expression of TJ proteins and functional TJs seems to indicate a possible active role of the latter in the keratinization process [42]. 

We observed a significant increase in the number of TJ remnants in skin explants compared to fresh skin biopsies. It was tempting to explain such a difference by the stress exerted on skin barrier during harsh antisepsis procedures and surgery. The 24 h delay between skin excision and fixation was apparently enough to reveal reactive modifications induced in the TJ system, which resulted in quantitative changes in the first two cornified layers of the explant skin. 

Chemical challenge with LSA, disturbing the SC structure, appears to induce a response in the living epidermal layers in terms of upregulation of TJ structures. Human keratinocytes in culture show increased proliferation and turnover when compared to native skin. Therefore, they have significantly more chances than those in skin explants to alter the expression of newly elaborated TJs in a way that would remain visible in the lower horny layer after a 24 h chemical challenge [41,42,43,44,45]. This may be one of the reasons why the SC of explants did not show a reactive increase in the constitution of TJs after topical exposure to 1% LSA. Another possibility is that the native SC, which provides a better barrier function than SC elaborated by keratinocytes cultured at an air/liquid interface [46,47], can better resist short-term chemical challenge. This would suggest, in turn, that living epidermis is capable of sensing topical aggression, as in the case of the leaky SC barrier, and reacts by compensatory overexpression of the secondary outside-in permeability barrier of TJs. Our observation supports that of Baek et al., who reported a TJ response to the abrogation of the primary SC barrier in nude mice [48]. Acute abrogation of the SC barrier by tape stripping or acetone treatment has been shown to dissipate the epidermal calcium ion gradient, resulting in a massive burst of lipid extrusion at the top of the denuded granular layer, a mechanism that re-establishes relative surface waterproofness [49]. Interestingly, TJs may play a role in the recovery of the epidermal calcium gradient and, therefore, the terminal differentiation of keratinocytes and SC formation [50].

Our observations of pathological, hyperkeratotic epidermis indicate the role of TJ-derived structures in the compensatory upregulation of SC cohesiveness. In line with our above -mentioned experimental data, the impaired SC barrier formation observed in both studied genodermatoses also resulted in a notable increase in the number of cross-linked TJ remnants. Although the pathomechanisms of the studied diseases differ strikingly, one being related to TJ dysfunction and the other to corneodesmosome fragility, the reactive hyperkeratotic response was revealed to be similar in terms of TJ overexpression. 

The increased presence of TJ remnants riveting corneocytes laterally and subdividing the SC extracellular space is likely to result in (1) enhanced direct cohesion in the newly formed SC barrier and (2) reduction of accessibility of the extracellular hydrolytic enzymes to corneodesmosomes, which potentially delays desquamation. 

We suggest that such an upregulation of TJ expression is a dynamic compensatory response to the disruption of the principal permeability barrier of the SC. Quantitation of TJ remnants in the SC is a reliable ultrastructural method permitting evaluation of the capacity of epidermis to respond to changes in SC permeability. It helps us better understand the dynamics of pathophysiological changes occurring in living epidermal layers.

Differences in the epidermal permeability barrier related to body region do occur and are related to SC thickness, pilosity, and the local abundance of secretory glands [51]. Nevertheless, we did not detect any differences between the numbers of TJ remnants observed in abdominal and arm skin, suggesting that similar TJ homeostasis is in play in both these body sites. 

Regulation of expression and function of TJs largely depends on their composition, namely, the presence and assembly of several TJ proteins. Experimental and treatment-oriented modulation of the epidermal TJ system can therefore be exploited [52,53,54,55,56,57,58,59,60]. Alteration of expression of TJ components modifies permeability, even though the junction structures may persist morphologically [12]. A decrease in expression of transmembrane TJ proteins has been reported in various organs of aged individuals. In our studies, cells and tissues used for comparisons, RHE and skin explants, whether or not chemically treated, belonged to the same age groups. Therefore, age-influenced differences in the expression of TJ proteins could not explain the observed results. Specimens of pathological skin, obtained in children and young adults, were compared with a control group of young-to-middle-aged persons (not including any old people), in order to avoid any age-dependent bias. In the process of cornification, TJ structures are immobilized at the periphery of corneocytes in the nascent SC. These cell–cell contacts become cross-linked within the cornified envelopes, independent of their initial protein composition, as do desmosomes [25]. However, contrary to corneodesmosomes which show prominent intercellular cores, the fused lipid membranes of former TJs show little space for the action of the extracellular proteolytic enzymes involved in SC desquamation. Consequently, such TJ remnants possess none of the dynamic functions of TJs found in the SG. Instead, they provide enzyme-proof rivets, the number and positioning of which may influence the turnover of SC. 

It seems that both epidermal permeability barriers are inter-dependent and the modulation of one impacts the other. Changes in the integrity of the SC induce rapid activation of the highly dynamic system of TJ assembly, whereas experimental modification of the TJ system in the SG has less visible, immediate impact due to the slow pace of SC constitution. Nevertheless, traces of the changes induced do persist in the horny layer, in the form of TJ-derived corneocyte fusions, and impact SC dynamics.

## 4. Material and Methods

### 4.1. Skin Samples from Healthy Volunteers and Patients

All human samples were harvested according to the principles of the Helsinki declaration and collected in biobanks approved by local ethics committees. 

### 4.2. Electron Microscopy

Existing blocs of normal and pathological human skin and RHE were used in this study. All biopsies and tissue culture fragments were proceeded following standard electron microscopy (EM) procedures. Briefly, tissues were fixed in buffered 2.5% glutaraldehyde and post-fixed in 1% osmic acid. Alcohol-dehydrated fragments were embedded in epoxy resins. Ultrafine sections, counterstained with lead citrate and uranyl acetate, were examined with a transmission electron microscope at 100 kV (Philips CM120, Eindhoven, The Netherlands). All chemical compounds mentioned in the present section were obtained from Sigma-Aldrich (Saint-Quentin-Fallavier, France).

### 4.3. Skin Explants

NHS fragments issued from surgical remnants of breast reduction and abdominoplasty were cleared of adipose tissue and maintained at 32 °C on humid gauze impregnated with Hanks’ minimum essential medium (Gibco^®^, Thermo-Fisher Scientific, Illkirch-Graffenstaden, France). Glass or inox chambers (closed or open) were applied to the skin surface and were filled or not with the tested solutions for 24 h.

### 4.4. RHE

In vitro reconstructed human epidermis, EpiSkin^®^, was obtained from Episkin, Lyon, France and cultured according to the manufacturer’s instructions. The chosen topical treatment was applied using apposable inox rings. 

Beta-lipohydroxy salicylic acid (LSA) developed by l’Oréal (Aulnay sous Bois, Paris, France) was a generous gift from the manufacturer.

### 4.5. Quantitation of TJ Remnants

“Fused” morphology of inter-corneocyte contacts (as visualized in Figure 1c) was recorded and the occurrence of such structures quantified. The number of “fused” points of contact observed at the apical-lateral parts of the cells composing SC compactum was reported as a percentage of all lateral contacts visualized in a given sample (at least 100 spots on numerous ultrafine sections). In biopsies of pathological skin, quantitation was extended to several layers of hyperkeratotic SC, as mentioned in the appropriate tables.

### 4.6. Statistical Analysis

Statistical comparison was performed using a Chi^2^-test (χ² test).

## 5. Conclusions 

TJs in human epidermis:

- Constitute an additional permeability barrier, beneath the SC;
- Are involved in the process of formation of the SC barrier;
- Are mostly fragmented but may be up- or downregulated in SG;
- Their cross-linked TJ remnants participate in the SC cohesion and impact desquamation.

Modulation of the epidermal TJ may have impact on:

- The process of terminal differentiation and cornification;
- The persistence of TJ remnants in the SC and, thus, on desquamation;
- The permeability of the epidermal barrier.

## Figures and Tables

**Table 1:** TJ-like points of fusion in the lowermost SC (SC compactum) of normal human skin.

Skin Source	Additional Pretreatment	% of Apical-Lateral Intercorneocyte Contacts Showing Close Apposition (Fusion) of the Lipid Envelopes (χ² Test)	
**Normal skin explants** (abdominoplasty; 24 h, 37 °C)	**non-occluded** (*n* = 5)	**64.26**	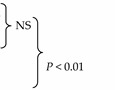
	**occluded** (*n* = 5)	**59.14**	
**Fresh skin biopsies** (arm)	**non-occluded** (*n* = 20)	**40.15 ± 6.11**	

NS = not significant.

**Table 2:** Topical chemical aggression in vitro (RHE), but not ex vivo (explants), induces an increase in TJ-derived fusions in the nascent SC. TJ-derived fusions within the apical-lateral contacts of corneocytes were counted in the first two layers of SC after 24 h topical treatment with beta-lipohydroxy salicylic acid (LSA). At least 100 apical-lateral contacts were examined at high magnification on several ultrafine sections of each specimen. NS = not significant.

	Vehicle	1% LSA	(χ² test)
Skin explants ex vivo (n = 10)	44% ± 8	48% ± 12	NS
Reconstructed epidermis RHE (n = 10)	57% ± 6	70% ± 6	*p* < 0.05

**Table 3:** Increased presence of fused TJ remnants in the SC of IHSC. TJ-derived fusions within the apical-lateral contacts of corneocytes were counted in the first four layers of SC. At least 100 apical-lateral contacts were examined at high magnification on several ultrafine sections of each specimen. NHS = normal human skin.

SC 1–4	% “Fused” Apical-Lateral Contacts
**IHSC** patient 1	70.4
**IHSC** patient 2	59.8
**NHS** (n = 35)	51.5 ± 7.6

**Table 4:** Fraction of the apical-lateral contacts between corneocytes showing “fused” morphology in the SC of PSD. NHS = normal human skin.

SC 1–4	% “Fused” Lateral-Apical Contacts
**PSD** patient P2	79.2
**PSD** patient P3	74.0
**NHS** (n = 35)	51.5 ± 7.6

## Data Availability

Data reported in this study were collected and stored by MH.

## Supplementary Materials

The following supporting information can be downloaded at: https://www.mdpi.com/article/10.3390/ijms23137486/s1.
