# Supplementary material for: The Fate of Epidermal Tight Junctions in the stratum corneum: Their Involvement in the Regulation of Desquamation and Phenotypic Expression of Certain Skin Conditions
Source: Int J Mol Sci. 2022 Jul 5;23(13):7486. doi: 10.3390/ijms23137486 (PMC9267462; doi:10.3390/ijms23137486)
Supplement: Supplementary file 1 [file ijms-23-07486-s001.zip › ijms-1768699-supplementary.pdf]

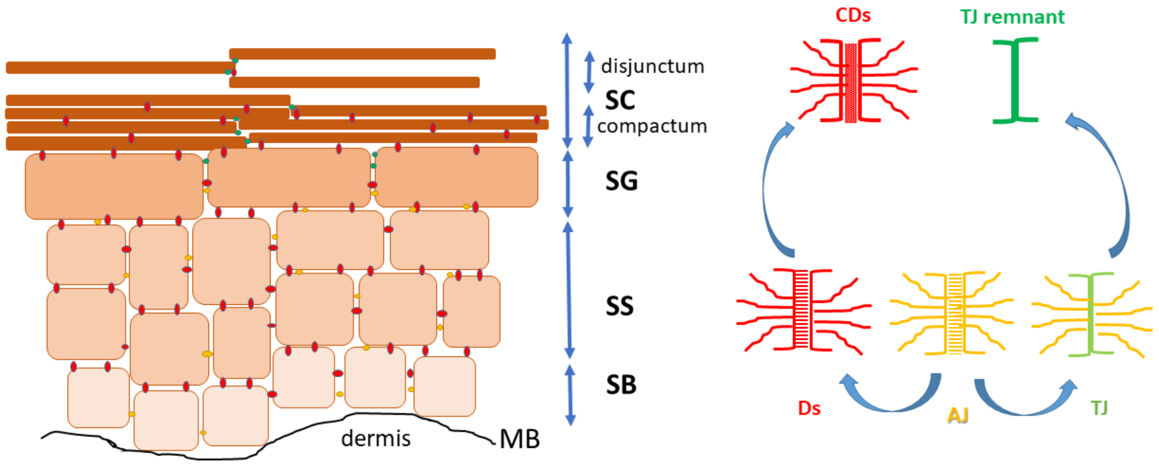

Supplementary Figure S1. Epidermis (left panel) and its 'mechanical' junctions (right panel). Epidermis is composed of keratinocytes arranged in several layers: stratum basale, SB, stratum spinosum, SS, stratum granulosum, SG, and stratum corneum, SC. Keratinocytes divide in the basal layer and differentiate to finally form a protective barrier composed of dead, fully cornified cells (corneocytes) at the top of the skin. Desmosomes (Ds) are the principal mechanical junctions keeping epithelial cells together. Their transmembrane cadherins (in red) are associated with keratin cytoskeleton inside the cells. In the SC, these junctions express additionally corneodesmosin in the extracellular gap and are called corneodesmosomes, CDs. Proteolytic degradation of these junctions is a morphological marker and leads to desquamation. The SC may be thus subdivided to the SC compactum, where CDs are abundant and the SC disjunctum, with only scarce CDs. Formation of Ds in the living layers of the epidermis is preceded by establishment of labile cell-cell contacts, adherens junctions (AJ), through classical cadherins associated with the actin cytoskeleton (in yellow). AJ are also a starting point of formation of tight junctions (TJ, in green). TJ are elaborated in SG through a very intimate association of specific transmembrane proteins (occludin, claudins, JAMs…), leaving no visible extracellular space. During the process of cornification, CDs and TJ become cross-linked at the periphery of corneocytes. For a more detailed description, please consult textbooks (2).
BM = basement membrane.

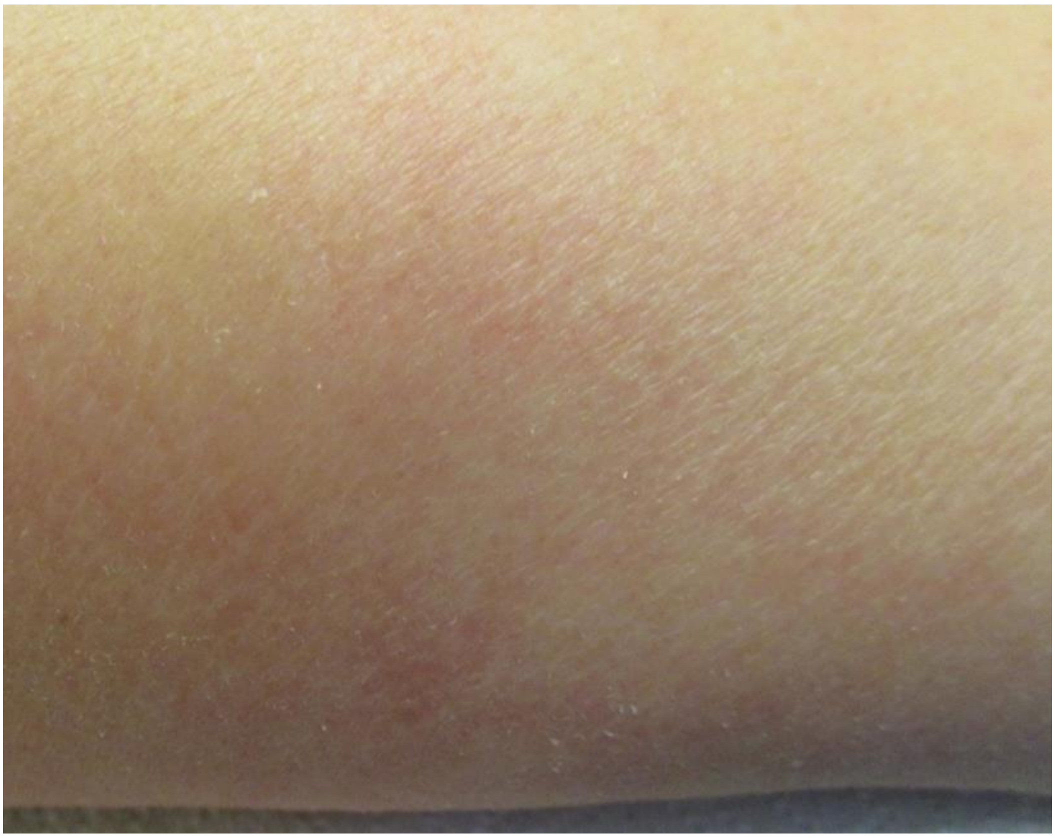

Supplementary Figure S2: Clinical picture of IHSC skin (patient #2). Ichthyosis is mild, giving a shiny aspect of compact, laminated SC surface.

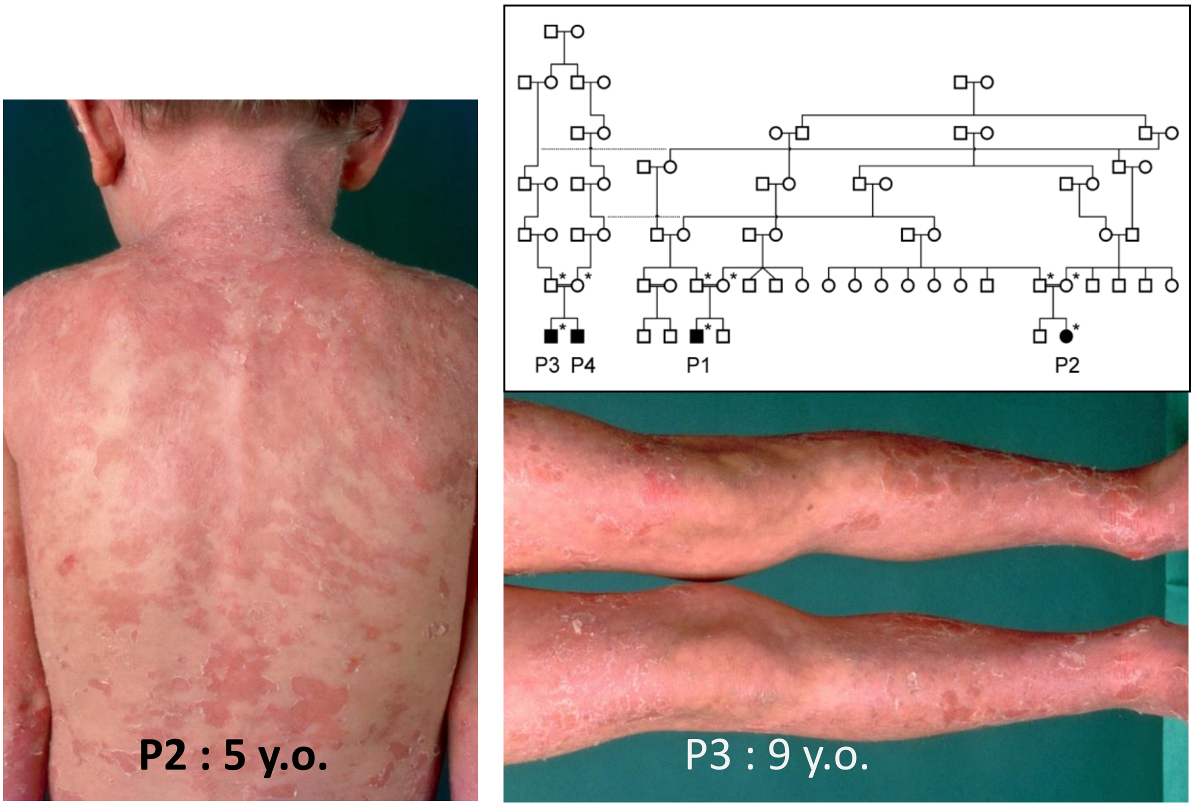

Supplementary Figure S3: Superficial detachment of SC following minor trauma in the studied PSD patients and their family relationships. The family 'tree' with indicated patients P2 and P3, included in the present study.
